# Calcium-Doped Boron Nitride Aerogel Enables Infrared Stealth at High Temperature Up to 1300 °C

**DOI:** 10.1007/s40820-021-00754-9

**Published:** 2021-12-06

**Authors:** Mengya Zhu, Guangyong Li, Wenbin Gong, Lifeng Yan, Xuetong Zhang

**Affiliations:** 1 School of Nano-Tech and Nano-Bionics, University of Science and Technology of China, Hefei, 230026 People’s Republic of China; 2 Suzhou Institute of Nano-Tech and Nano-Bionics, Chinese Academy of Sciences, Suzhou, 215123 People’s Republic of China; 3 School of Physics and Energy, Xuzhou University of Technology, Xuzhou, 221018 People’s Republic of China; 4 School of Chemistry and Materials Science, University of Science and Technology of China, Hefei, 230026 People’s Republic of China; 5 Division of Surgery & Interventional Science, University College London, London, NW3 2PF UK

**Keywords:** Aerogel, Boron nitride, Calcium doping, Infrared stealthy, Butane flame

## Abstract

Boron nitride (BN) aerogels, composed of nanoscale BN building units together with plenty of air in between these nanoscale building units, are ultralight ceramic materials with excellent thermal/electrical insulation, great chemical stability and high-temperature oxidation resistance, which offer considerable advantages for various applications under extreme conditions. However, previous BN aerogels cannot resist high temperature above 900 °C in air atmosphere, and high-temperature oxidation resistance enhancement for BN aerogels is still a great challenge. Herein, a calcium-doped BN (Ca-BN) aerogel with enhanced high-temperature stability (up to ~  1300 °C in air) was synthesized by introducing Ca atoms into crystal structure of BN building blocks via high-temperature reaction between calcium phosphate and melamine diborate architecture. Such Ca-BN aerogels could resist the burning of butane flame (~  1300 °C) and keep their megashape and microstructure very well. Furthermore, Ca-BN aerogel serves as thermal insulation layer, together with Al foil serving as both low-infrared-emission layer and high-infrared-reflection layer, forming a combination structure that can effectively hide high-temperature target (heated by butane flame). Such successful chemical doping of metal element into crystal structure of BN may be helpful in the future design and fabrication of advanced BN aerogel materials, and further extending their possible applications to extremely high-temperature environments.

**Supplementary Information:**

The online version contains supplementary material available at 10.1007/s40820-021-00754-9.

## Highlights

- A calcium-doped boron nitride (Ca-BN) aerogel was synthesized by introducing Ca into crystal structure of BN building blocks.
- The aerogels exhibited superior high-temperature stability and could resist the burning of butane flame (~1300 °C) in air atmosphere.
- Ca-BN aerogel, together with Al foil, can effectively hide thermal target at high temperature up to 1300 °C.

**Supplementary Information:**

The online version contains supplementary material available at 10.1007/s40820-021-00754-9.

## Graphical abstract

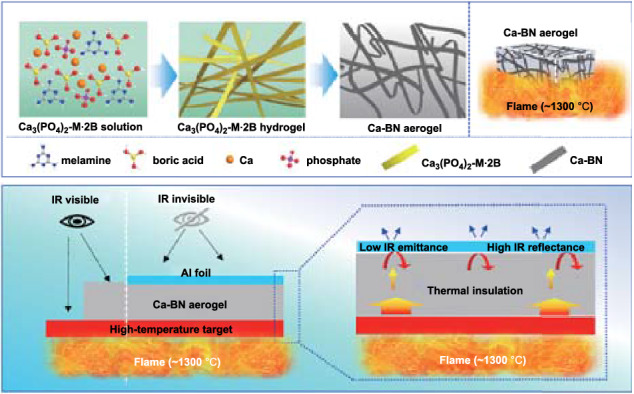

## Introduction

Boron nitride (BN) aerogels are the ultralight ceramic materials assembled by BN nanoscale building block [1–3], and they exhibit low density, large specific surface area, excellent thermal/electrical insulation, outstanding chemical stability and desirable high-temperature oxidation resistance, which offer considerable advantages for various applications under extreme conditions [3], such as high-temperature superinsulation, thermal protection and environmental cleaning in harsh environment (acid, alkali), while the polymeric and carbonaceous aerogels could collapse easily with performance damping [4–7]. During the process of BN aerogels, how to construct porous network with nanoscale skeleton using BN nanoscale building blocks is of great significance for the mainstay of various applications [1]. Unfortunately, most of pristine BN nanoscale building blocks were fabricated by CVD growth or exfoliation from bulky BN, and they involve hazardous chemicals and arduous procedures [8, 9]. Moreover, the pristine BN nanoscale building blocks exhibited instinct chemical inertness and poor solution chemistry [8, 10, 11], so the traditional molecular sol–gel process that utilizes hydrolysis–condensation of small precursor solution cannot fit in well with the BN aerogel [12, 13], which further limit BN aerogels to very few varieties and hinder their practical applications.

To add the diversity of BN aerogel microstructures and unlock their applications, various specific synthetic strategies that are different to the traditional sol–gel process have been explored [1–3], and various building blocks with different dimension were successfully used to construct BN aerogel porous network, such as nanoribbon [14, 15], nanotube [16], nanosheet [17] and micro-/nanofiber [18]. Briefly, Rousseas et al*.* developed a BN aerogel with highly chemical purity via the carbothermal reduction reaction between graphene aerogel, boron oxide and nitrogen, and this aerogel exhibited a unique interlinking structure [2]. Xu et al*.* designed a BN aerogel with the hyperbolic architecture through a graphene aerogel-assisted chemical vapor deposition process, and this BN aerogel exhibited double-negative-index (negative Poisson’s ratio and negative linear thermal expansion coefficient), which could serve as an ideal insulator for thermal superinsulation under extreme conditions [3]. However, most of the previous BN aerogels still cannot resist high temperature above 900 °C in air atmosphere, and the intrinsic chemical inertness of BN makes it impossible to enhance the high-temperature stability via chemical doping or modifying in solution. Therefore, the design and synthesis of advanced BN aerogels which can be applied at high temperature above 900 °C in the air are still a great challenge.

Herein, a calcium-doped boron nitride (Ca-BN) aerogel with enhanced high-temperature stability was fabricated through high-temperature treatment of calcium phosphate-doped melamine diborate architecture. The aerogel was constructed by intertwined Ca-BN micro-/nanoribbons, and Ca atoms were successfully doped into the crystal structure of BN nanoscale building blocks. Benefited from the doping of Ca atoms, the resultant Ca-BN aerogel exhibited superior high-temperature stability (~ 400 °C higher than pure BN aerogel). To be specific, the Ca-BN aerogel could resist burning of butane flame (up to 1300 °C) and maintain the microstructure very well. Moreover, Ca-BN aerogel together with Al foil, forming a combined structure which can effectively hide high-temperature target (heated by butane flame). The successful chemical doping with metal element into crystal structure of BN may be helpful in the future design and fabrication of advanced BN nanostructures and relevant aerogel materials, and further extend their application in extreme conditions.

## Experimental Section

### Materials and Methods

#### Materials

Calcium chloride dihydrate (CaCl_2_·2H_2_O, powder, 99.0%), phosphoric acid (H_3_PO_4_, 99.0%, powder), boric acid (H_3_BO_3_, AR, 99.5%), melamine (C_3_N_6_H_6_, AR, 99.0%), triethylamine ((C_2_H_5_)_3_ N, 99.0%), ethanol (C_2_H_5_OH, 99.7%) and tertiary butyl alcohol (C_4_H_10_O, 99.7%) were purchased from Aladdin Company. Argon gas was purchased from Linde Group, Suzhou.

#### ***Synthesis of Ca***_***3***_***(PO***_***4***_***)***_***2***_*** Ionic Oligomer***

Ca_3_(PO_4_)_2_ ionic oligomer was prepared according to the method of the literature [19]: typically, dissolving 0.588 g calcium chloride dihydrate in 48 mL ethanol, dissolving 0.262 g phosphoric acid and adding a certain amount of triethylamine (molar ratio Ca: TEA = 1:20) in 32 mL ethanol. The subsequent quick mixing of these two solutions by stirring generates a transparent or semitransparent solution that contained Ca_3_(PO_4_)_2_ ionic oligomers within 30 min. Ca_3_(PO_4_)_2_ ionic oligomers were concentrated by the high-speed centrifugation and then redispersed in tertiary butyl alcohol (TBA) twice to remove impurities and then obtained the Ca_3_(PO_4_)_2_ ionic oligomers solution.

#### Synthesis of Calcium-Doped Boron Nitride Aerogel

Dissolve 0.989 g boric acid and 1.137 g melamine in 44 mL H_2_O and 38 mL TBA co-solvent system at 85 ~ 90 °C water bath under stirring within 30 min to obtain a transparent solution. Add the Ca_3_(PO_4_)_2_ ionic oligomers solution obtained in the previous step into the hot solution, and stir for a few minutes to get another mixing transparent solution (Ca_3_(PO_4_)_2_-M·2B solution). The hot mixing solution was cooled naturally under simultaneously ultrasonic treatment until get a white hydrogel precursor (Ca_3_(PO_4_)_2_-M·2B hydrogel). Subsequently, the hydrogel was freeze-dried to get a white aerogel-like precursor (Ca_3_(PO_4_)_2_-M·2B aerogel). Finally, the precursor was transferred to a horizontal quartz tube and heated to 1400 °C for 3 h in the presence of argon gas, and a white calcium-doped boron nitride aerogel (Ca-BN aerogel) was obtained.

### Characterization

The morphology of the samples was characterized by field emission scanning electron microscope (SEM) (S-4800, Hitachi Company, Japan) operated at 10–20 kV, and Ca-BN aerogel was coated with Au sputtering under current of 25 mA for 2 min. Field emission transmission electron microscope (TEM) was carried out on Tecnai G2 F20 STWIN (FEI Company, USA) with the acceleration voltage of 200 kV. STEM and element mapping were collected by spherical aberration-corrected TEM (FEI Themis Z). The pore structure of the aerogels was investigated using a surface area analyzer (ASAP 2020 HD88, Micrometrics, Inc., USA). The Brunauer–Emmett–Teller (BET) method and the Barrett–Joyner–Halenda (BJH) model were utilized to calculate the BET specific surface area (SSA) and the pore size distribution.

The crystal structure of the as-prepared samples was investigated by X-ray diffraction (XRD, D8 advance, Bruker AXS). Raman spectroscopic investigation was carried out by laser confocal Raman spectrometer (LABRAM HR, Horriba-JY). The XPS spectra were measured by Escalab 250Xi, Thermo Scientific. Thermal stealthy and relevant IR images were recorded by Escalab 250 Xi, Thermo Scientific. The room temeprature thermal conductivity of samples was measured by transient hot wire method (XIATECH TC3000, China), and the data were collected five times with a 5-min interval between each measurement. TG and high-temperature thermal conductivity were measured via BioEngX. TG was carried out using NETZSCH STA 449F3. High-temperature thermal conductivity was carried out using Hot disk (base on international standard ISO 22007–2). Temperature detection was carried by IR thermometer (ISR 6 Advanced, LumaSense IMPAC).

### Simulation

Simulations combined molecular dynamics (MD) and density functional theory (DFT) were performed to investigate the structures of the Ca_3_(PO_4_)_2_-M·2B precursor and Ca-BN samples. The MD calculations were performed in the canonical ensemble at 300 K by using LAMMPS (large-scale atomic/molecular massively parallel simulator) code. The DREIDING forcefield was employed to describe the inter- and intra-interactions of Ca_3_(PO_4_)_2_ and M·2B molecules, while the atomic charges were calculated with the charge equilibration (QEq) method. The DREIDING forcefield was chosen because its validity in simulating the M·2B-related systems had been checked before. The structures of Ca-BN were investigated by performing the plane-wave-based DFT method implemented in the VASP (Vienna Ab Initio Simulation Package) code. A 6 × 6 × 1 bilayer h-BN with a 20 Å vacuum region, whose Brillouin zone was sampled by a 3 × 3 × 1 k-mesh, was constructed for all the simulations. A Ca atom was introduced into the model by either intercalating into the h-BN or substituting the B or N atoms of the h-BN. The electron ion interaction was described with the projector augmented wave method with the Perdew–Burke–Ernzerhof potential for the exchange–correlation functional. The cutoff energy was set to be 400 eV. The long-range van der Waals interactions were calculated with the Tkatchenko and Scheffler scheme, while the self-consistent screening and polarizability contraction effects were also considered, in view of their important roles in determining the weak intermolecular interactions of h-BN. The geometry optimization was performed when the convergence criterion on forces became smaller than 0.02 eV Å^−1^ and the energy difference was set to be 10^–5^ eV.

## Results and Discussion

### Characterization of Ca-BN Aerogel

For the pure BN, it is easy to oxidize into boric oxide and then evaporate rapidly in high-temperature (above 900 °C) oxidative environment [3, 14]. To improve the high-temperature oxidation resistance of BN aerogel material, it is essential to reduce the evaporation of boric oxide, and then form a fixed molten film structure on the surface of BN nanoscale building blocks. It has been known that the metallic oxide can react with melted boric oxide and generate an infusible or involatile compound (e.g., xCaO·yB_2_O_3_ system) during glass process [20–22]. Inspired by the production process of glass in the industry, the Ca atoms were introduced into the crystal structure of BN through the high-temperature treatment of calcium phosphate-doped melamine diborate (M·2B) architecture.

**Scheme 1:**
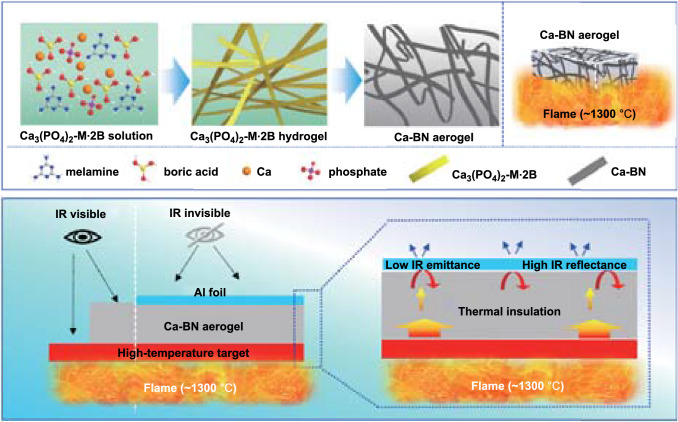
**a** Schematic synthesis of Ca-BN aerogels. **b** Scheme of high-temperature stabilization of Ca-BN aerogel. **c** Scheme of high-temperature infrared stealthy of Ca-BN aerogel together with Al foil at high temperature up to 1300 °C

**Fig. 1:**
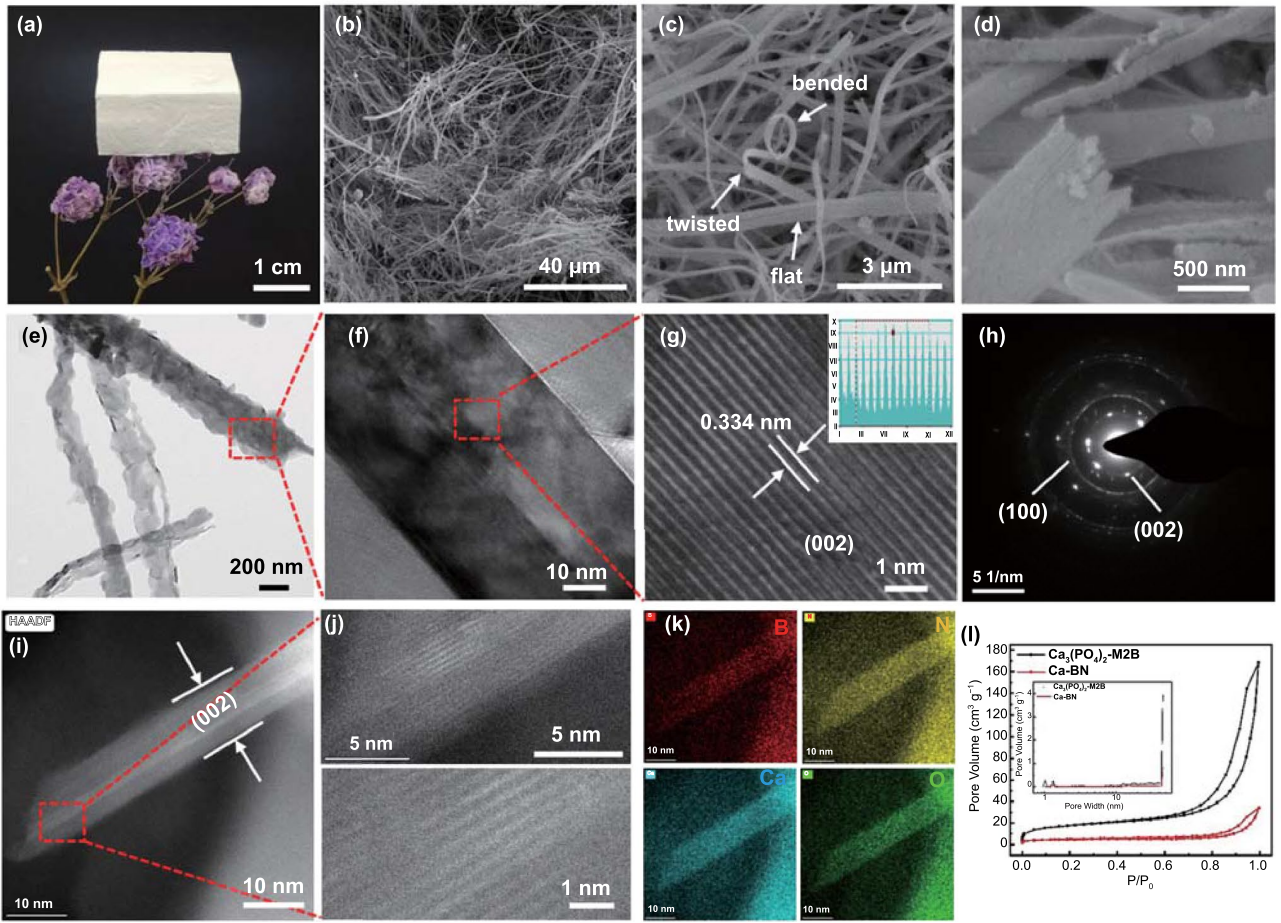
**a** Optical image of Ca-BN aerogel. **b**–**d** SEM images of Ca-BN aerogel. **e**–**f** TEM images of Ca-BN aerogel. **g**–**h** HRTEM image and the corresponding electron diffraction pattern of Ca-BN aerogel. **i**–**j** STEM images of Ca-BN aerogel. **k** STEM-EDS mappings of Ca-BN aerogel. **l** Nitrogen adsorption–desorption isotherm curves of Ca-BN aerogel and Calcium phosphate-doped M·2B precursor. Inset in image **l**, the corresponding pore size distribution curves

As shown in Scheme 1a and Fig. S1, the melamine, boron acid and calcium phosphate oligomer [19] were added into the hot water/tertiary butanol (TBA) co-solvent in sequence, and they formed a transparent and uniform mixed solution, and then, the solution is cooled to ca. 30 °C under the assistance of ultra-sonication to obtain a white hydrogel; after freezing-drying and high-temperature treatment (1400 °C, Ar atmosphere), the final Ca-BN aerogel with low density (20.41 mg cm^−3^) and white appearance was obtained (Fig. 1a). The melamine and boric acid have long been recognized as cheap and common precursors for BN [1], and they could assemble into melamine diborate (M·2B) nanoscale building block via the hydrogen bonding in the water/TBA co-solvent [14], while calcium phosphate could link to amine group (e.g., triethylamine, ethanediamine, melamine molecules) via hydrogen bonding and this has been reported elsewhere [19]. According to hydrogen bonding interaction, calcium phosphate will embed into the crystal structure of M·2B, which provides opportunity to dope Ca atoms into crystal structure of BN nanoscale building blocks, and results in a Ca-BN aerogel with enhanced high-temperature stability (Scheme 1b). Furthermore, Ca-BN aerogel serves as thermal insulation layer, together with Al foil serving as both low-infrared-emission layer and high-infrared-reflection layer, forming a combination structure that can effectively hide high-temperature target (Scheme 1c).

The morphology of Ca-BN aerogel was investigated by SEM. As shown in Fig. 1b-d, large amounts of micro-/nanoribbons intertwined and interweaved with each other to form a porous network, and cross sections of micro-/nanoribbons exhibit whisker-like structure with the irregular end and rough surface, resembling to the calcium phosphate-doped M·2B precursor (Fig. S2). The porous structure was further quantified by nitrogen sorption (Fig. 1l), and the isotherm curve of Ca-BN aerogel possessed a rapid increase in the low-pressure range (P/P_0_ < 0.02) and a hysteresis loop, which implied the existence of micropores and meso-pores in Ca-BN aerogel. According to Barrett–Joyner–Halenda (BJH) analyses, the specific surface area was about 14.7 m^2^ g^−1^.

**Fig. 2:**
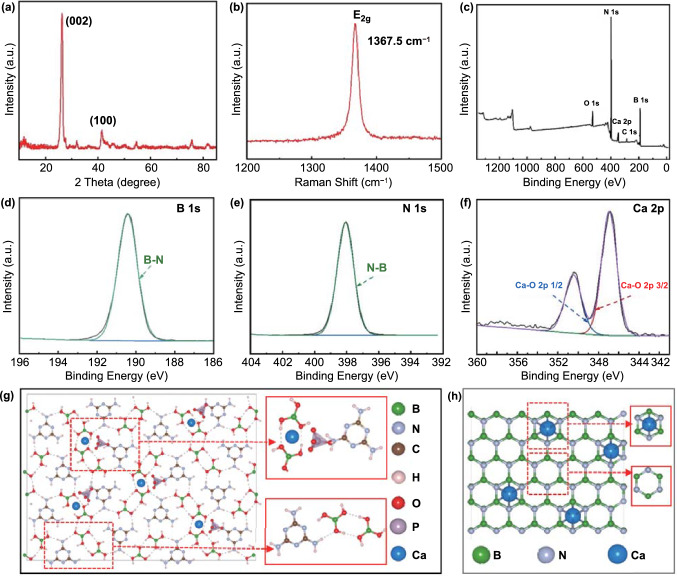
**a** XRD pattern of Ca-BN aerogel. **b** Raman spectrum of Ca-BN aerogel. **c** XPS survey of Ca-BN aerogel. **d**–**f** B 1s, N 1s, Ca 2p spectra of Ca-BN aerogel. **g** Schematic plots of Ca_3_(PO_4_)_2_ molecule adsorbed on M·2B. **h** Schematic plots of Ca atom in Ca-BN, the insets illustrate the detailed structure

Furthermore, the microstructure of single Ca-BN micro-/nanoribbon was recorded by TEM images (Figs. 1e, g and S3, S4). It is interesting to note that micro-/nanoribbons consist of many stripe-like and overlapped nanocrystals, most of these nanocrystals were distributed throughout micro-/nanoribbon and some of them are dendritic crystals hang on the edge of micro-/nanoribbon; as shown in Figs. 1f and S3, S4, the size of single nanocrystal could reach to hundreds of nanometers (Fig. 1f, g), which is different from the amorphous structure in previous BN nanoribbon aerogel without chemical doping (Fig. S5) [14]. Moreover, the clear lattice fringes were observed in the high-resolution TEM image and they displayed a typical layer spacing of ~ 0.334 nm (Fig. 1f, g), which is consist with that of bulk h-BN (0.333 nm, Fig. S6); such results were further confirmed by the selected area electron diffraction patterns (Figs. 1h and S4) [18, 23], XRD (Figs. 2a and S6, S7) and Raman (Fig. 2b) spectra. Typically, Ca-BN aerogel exhibited high crystalline similar to pristine h-BN, while BN aerogel derived from M·2B monolith was amorphous (Fig. S6).

To investigate and confirm the existence of Ca in aerogel, the element mappings of single nanocrystal (crystal plane, 002) with clear lattice fringes (Figs. 1i, j and S8, S9) were recorded firstly. B, N, O and Ca elements are uniformly distributed along and across the single nanocrystal (Fig. 1k), but phosphorous (P) element cannot be detected (Fig. S9). It is speculated that in the process of high-temperature annealing, the calcium phosphate occurred carbothermal reduction reaction with carbon and boric oxide, and then, phosphorus volatilized in the form of phosphorus oxide. Notably, as element signals were collected on the lattice fringe of typical (002) crystal face of BN, no other crystal or amorphous structures were observed, and Ca element distributed in this crystal plane, indicating Ca element was dopped into the crystal structure of BN.

The chemical compositions were further confirmed by X-ray photoelectron spectroscopy (XPS) spectra (Figs. 2c, f and S10-S12), the B, N, C, O and Ca elements were detected in the Ca-BN aerogel, and P element still cannot be detected, which consists to the results of the above element mapping (Figs. 1k and S9). Furthermore, both high-resolution XPS B 1 s and N 1 s spectra can be fitted into one peak at 190.4 (Fig. 2d) and 398.1 (Fig. 2e) eV, respectively, which confirms the formation of BN. For Ca 2p spectrum (Fig. 2f), and there are two fitted peaks located at 346.9 and 350.4 eV, corresponding to 2p_3/2_ and 2p_1/2_ (generated by the spin–orbit splitting), respectively, which can be assigned to Ca–O. O 1 s spectrum also shows the Ca–O bond at 531.5 eV (Fig. S12), indicating Ca element existed as Ca–O compound in Ca-BN aerogel (Figs. S10-S12).

To investigate the role of calcium phosphate oligomer in fabrication process of Ca-BN aerogel, calcium phosphate oligomer solution was freeze-dried firstly, and the obtained solid consisted of large amounts of irregular particles and pieces (Fig. S13). However, such irregular or flaky morphologies were not found in calcium phosphate-doped M·2B precursor (Fig. S2). If calcium phosphate oligomer was replaced by commercial calcium phosphate powder and used to fabricate calcium phosphate/M·2B composite precursor, the obtained precursor monolith exhibited lots of micron-sized particles in the porous network constructed by ribbons (Figs. S14 and S15). These different morphologies of the above samples provided preliminary evidence that the calcium phosphate oligomer is essential to this fabrication and it was embedded into M·2B ribbon. Moreover, as calcium phosphate oligomer was introduced into M·2B units, the obtained ribbons exhibited a typical whisker-like structure in their cross section (Figs. 1d and S16), indicating that these ribbons were consisted of large amounts of banded ligatures, which is different from the nanoribbons of pure M·2B nanoribbon (smooth hexagonal end) in previous reports [1, 14].

To understand the existing form of Ca element in aerogel, simulations combined molecular dynamics (MD) and density functional theory (DFT) were performed. It is found that the Ca_3_(PO_4_)_2_ molecule is adsorbed on the M·2B precursor through electrostatic and H bonding interaction as shown in Fig. 2g, without destroying the M·2B network. As a result, Ca_3_(PO_4_)_2_ molecule could serve as an excellent binder to connect the adjacent M·2B sheets in the solvent. With the high-temperature treatment, the Ca-BN sample was obtained. The Ca atom will either substitute a B/N atom in a single layer or exist in the space between the adjacent Ca-BN sheets. DFT simulations indicate that the Ca substitution of B or N atom leads to a narrowed interlayer distance (d) about 3.14 Angstrom compared with raw Ca-BN with the d about 3.31 Angstrom, with the intercalation of Ca atom in Ca-BN results in a slightly expanded d about 3.36 Angstrom. Since the observed interlayer distance d in the experiment is about 3.4 Angstrom, the Ca atom should be existed in h-BN with the intercalation form as shown in Fig. 2h.

Mechanical property of Ca-BN aerogel was investigated by compress test (Fig. S17). The stress–strain curve exhibited three deformation regimes: an initial Hookean region (elastic deformation) at strain ε < 8% with a Young’s modulus of about 25 kPa, a plateau (plastic deformation) and the final densification region. In addition, as Ca-BN aerogel was compressed to different strains, respectively, ε from 5 to 25% with an interval of 5%, it can recover to original shape (Fig. S17b), indicating Ca-BN aerogel was mechanical elasticity.

### High-Temperature Stability of Ca-BN Aerogel

**Fig. 3:**
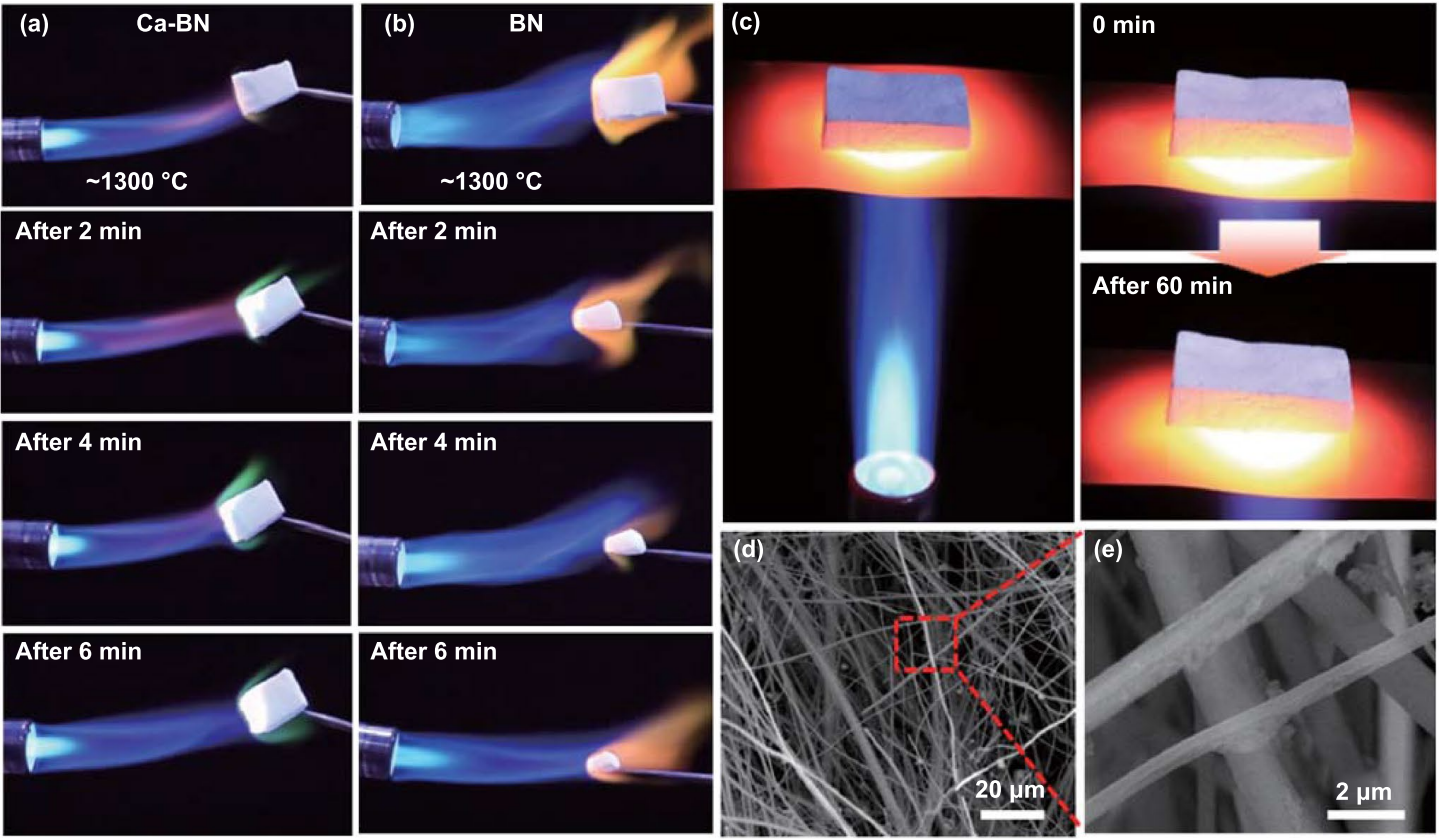
**a**–**b** Optical images of Ca-BN aerogel and pure BN aerogel heated by butane blowtorch flame. **c** Ca-BN aerogel monolith was placed on the red-hot steel foil which heated by butane flame in air for 1 h. **d-e** SEM images of bottom surface after heated for 60 min

**Fig. 4:**
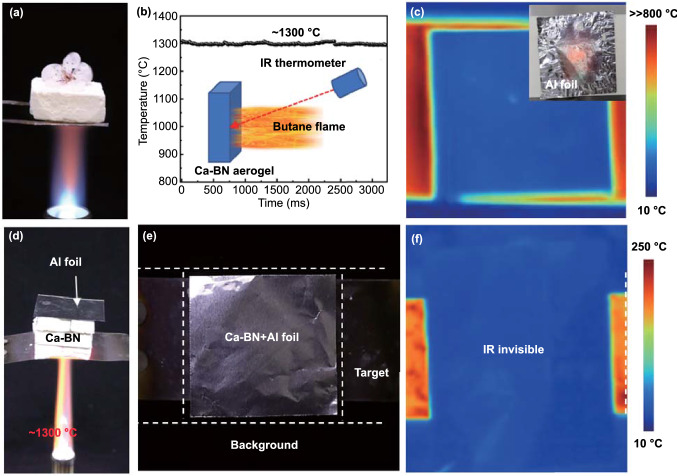
**a** Photograph of the high-temperature thermal insulation test of Ca-BN aerogel. Ca-BN aerogel effectively insulates the flower against the fire with temperature up to ~  1300 °C. **b** Temperature–time curves of Ca-BN aerogel which heated by butane flame. **c** IR image of hot target covered by Al foil, and the target was heated by butane flame. **d**–**f** Optical images (side view and top view) and IR image of hot target covered by Ca-BN aerogel and aluminum foil

To investigate the high-temperature oxidation resistance performance of Ca-BN aerogel, a butane flame (~ 1300 °C) was used to burn Ca-BN aerogel for about 6 min, and the megashape of the white aerogel monolith maintained very well (Fig. 3a), while the pure BN aerogel monolith faded away and disappeared nearly invisible (Fig. 3b). Moreover, temperature of the burned surface of Ca-BN aerogel was detected by infrared thermometer, and it could reach to about ~ 1309 °C (Figs. 4b and S18), which provided a direct evidence to confirm the temperature could be up to 1300 °C. These different experiment phenomena between Ca-BN and pure BN aerogel mainly attributed to their different chemical composition. Under the burning of butane flame, pure BN aerogel was oxidized into B_2_O_3_ and then evaporated rapidly (Fig. S19). For the Ca-BN aerogel, the Ca element could react to melted B_2_O_3_ and then form an involatile melted compound (e.g., xCaO·yB_2_O_3_ system) [22], and the melted compound covered on the skeleton surface of the aerogel and prevented the further oxidization, as shown in Figs. 3d, e and S19, S20. Moreover, the mixed powder of boric acid and CaO was heated to about 1300 °C, and the powder becomes transparent solid which adheres on white substrate (Fig. S21), indicating the generation of involatile melted compound. In other words, the above results provided a strong and visual evidence to confirm the effect of Ca element in BN aerogels, i.e., Ca element enhanced the thermal stability and oxidation resistance performances of BN aerogel.

The high-temperature stabilization of Ca-BN aerogel was further investigated according to a unidirectional heating experiment in the air. As the Ca-BN aerogel monolith was placed on the red-hot steel foil which heated by butane flame (~ 1300 °C), these experiment design could eliminate the shocking of the butane flow generated by flamer (Fig. 3c). After heating for about 60 min, the megashape of aerogel monolith still maintained very well (Fig. 3c). Moreover, during heating process, size and mass of Ca-BN aerogel were recorded every 10 min, and the sizes along different directions, color and shape of aerogel monolith are consistent before and after high-temperature treatment (Figs. S20a and S23), while the mass only reduced slightly by less than 1.5 wt%. Under the similar heating condition, the size and mass of pure BN aerogel exhibited obvious change (Fig. S20b). Furthermore, after heating about 60 min, the bottom (heated) surface morphology was recorded by SEM images, the micro-/nanoribbon building blocks still could be observed, and there was no change in the overall porous network, although there are few hemispherical grains hung on the edge of ribbons (Fig. 3d, e). All these results further indicated that the Ca-BN aerogel possessed excellent high-temperature long-term stability in the air, and the Ca element contributed to such fascinating properties.

### High-Temperature Thermal Insulation

In addition to its superior high-temperature oxidation resistance performance, the Ca-BN aerogel still possessed excellent thermal insulation behavior. In general, the thermal conductivity mainly depends on three components: thermal convection, thermal conductivities of gases and solids, and heat radiation. The prepared Ca-BN aerogel with plenty of micro-/meso-pores possessed a low density that corresponding to a high pore porosity, which could efficiently mitigate the air conduction and convection. The randomly porous network intertwined by micro-/nanoribbon provided large amount interfaces that can result in a low solid thermal transmission. According to the transient hot wire method at room temperature, the Ca-BN aerogel exhibited a low thermal conductivity of 0.0454 ± 0.0028 W/mK, which is comparable to the previous ceramic aerogels and other porous materials, such as SiO_2_-Al_2_O_3_ composite sponges (0.034 W mK^−1^) [24], ZrO_2_-Al_2_O_3_ nanofibrous aerogels (0.032 W mK^−1^) [25] and pure BN aerogel (~ 0.035 W mK^−1^) derived from M·2B precursor [14]. Based on the above comparison, as the Ca element was introduced into BN aerogel, the thermal conductivity increased slightly, and this mainly attributed to the improved crystal structure of micro-/nanoribbons in the aerogel, which provided an adverse effect for phonon scattering. Moreover, the thermal conductivity of Ca-BN aerogel still lower than that of densified BN aerogel film (0.055 W mK^−1^) [15], BN nanocomposite aerogel (2.2 W mK^−1^) [17] and graphene aerogels (0.2–2.1 W mK^−1^) [26, 27].

High-temperature thermal conductivities of Ca-BN aerogel were further measured by a hot disk thermal analyzer, and their values were about 0.14 and 0.15 W mK^−1^ at 900 and 1000 °C, respectively, lower than that of most aerogel materials in the previous reports [28–32], such as Al_2_O_3_ aerogel (0.298 W mK^−1^ at 800 °C) [28], core–shell Al_2_O_3_ aerogel/mullite fiber composite (0.16 W mK^−1^ at 1000 °C) [29], palygorskite-based aerogels (0.165 W mK^−1^ at 1000 °C) [30], aluminum borate foams (0.228 W mK^−1^ at 1000 °C) [31] and BN/SiOC aerogel (0.215 W mK^−1^ at 1000 °C) [32]. Therefore, Ca-BN aerogel exhibits great potential in high-temperature insulation.

Combined with the high-temperature stability and low thermal conductivity, the Ca-BN aerogel could be used as thermal insulator under extreme conditions, such as high-temperature oxidation environment. As shown in Fig. 4a, the Ca-BN aerogel monolith can effectively protect the fresh flower from the withering or carbonization under the heating of butane flame. In addition, thermal insulation property was further investigated via infrared camera. As the Ca-BN aerogel monolith (3.0 cm thick) was placed on a corundum plate with high temperature, as shown in Fig. S24, a clear temperature gradient through the Ca-BN aerogel was observed. As the heating time increases, the top surface of the Ca-BN aerogel was maintained a nearly constant temperature at about 102.5 °C when thickness of Ca-BN aerogel was about 3 cm (Fig. S24).

### High-Temperature Thermal Stealthy

The extraordinary high-temperature stability and superior high-temperature heat insulation performance of the Ca-BN aerogel make it useful in high-temperature thermal stealthy [33–36]. According to the Stefan–Boltzmann law: P = εσT^4^, the thermal radiation of a target is proportional to the surface emittance (ε) and the fourth power of temperature (T) [33–37]. Thermal stealthy is need to blend targets into their surroundings in infrared imaging to evade infrared thermal detection. For this purpose, various materials and architectures were proposed reducing temperature or regulating IR emissivity of a target and then achieving thermal stealthy, but the realization of thermal stealthy at high temperature up to 1300 °C in the air still remains a great challenge.

For high-temperature thermal stealthy, superior high-temperature stabilization is necessary. As the Ca-BN aerogel was covered on the surface of high-temperature target, IR image of Ca-BN aerogel showed a similar color to the background, and as the thickness of BN aerogel increased, the contrast between aerogel surface and environment decreases and becomes almost consistent (Fig. S25), indicating the radiation temperature of high-temperature target (~ 1300 °C) was reduced significantly (reduced to about 100 °C). However, only relying on the thermal insulation of Ca-BN aerogel, the effect of thermal stealthy is limited, as the surface radiation temperature still larger than the background.

Integrating thermal insulation and desired infrared emission in one architecture, which can further reduce the radiation temperature of high-temperature target to around room temperatures, may provide a chance to achieve superior high-temperature thermal stealthy. Aluminum foil possessed a very low infrared emissivity and high infrared reflectance [29, 37–40], and it is usually used in thermal stealthy. However, the aluminum foil will be melted or destroyed in the high temperature above 660 °C environment and then brought safety problem or become infrared visible (Fig. 4c). Hence, a combination architecture between Ca-BN aerogel and aluminum foil was proposed for high-temperature IR stealthy (Fig. S26) [33–36]. As the aluminum foil was covered on the surface of Ca-BN aerogel, and they physically stacked with each other, the contract between target and background further decreased (Figs. S25 and S26), promoting the IR invisibility of high-temperature target. Typically, as the butane flame (~ 1300 °C, Fig. 4b) was used to heat the target, the combination structure of Ca-BN aerogel and aluminum foil was covered on the high-temperature region (Figs. 4d, e and S27), and the high-temperature target become completely infrared invisible (Figs. 4f and S28), as the radiation temperature detected by infrared camera was about 31 ± 2 °C, indicating that such Ca-BN aerogel can serve as a promising candidate for high-temperature thermal stealthy [33–36].

## Conclusion

In summary, a Ca-BN aerogel was fabricated through high-temperature treatment of calcium phosphate-doped melamine diborate architecture. This aerogel was constructed by intertwined Ca-BN micro-/nanoribbons, and the Ca element was embedded into the crystal of BN building blocks. Such structure provided an exciting stability, oxidization resistance and excellent thermal insulation at high temperature up to 1300 °C oxidation environment. Typically, this aerogel could resist the burning of butane flame and maintain the megaship very well. Moreover, the Ca-BN aerogel together with Al foil, via forming a combined structure, can effectively hide high-temperature target (heated by butane flame). The successful doping metal element into crystal structure of BN may be helpful in the future design and fabrication of advanced BN nanostructures and relevant aerogel materials, and further extend their applications in extreme conditions.

## Supplementary Information

Below is the link to the electronic supplementary material.

Supplementary file1 (PDF 1786 KB)
