# Supplementary material for: Calcium-Doped Boron Nitride Aerogel Enables Infrared Stealth at High Temperature Up to 1300 °C
Source: Nanomicro Lett. 2021 Dec 6;14:18. doi: 10.1007/s40820-021-00754-9 (PMC8649065; doi:10.1007/s40820-021-00754-9)
Supplement: Supplementary file 1 — Supplementary file1 (PDF 1786 KB) [file 40820_2021_754_MOESM1_ESM.pdf]

Supporting Information for

Calcium-Doped Boron Nitride Aerogel Enables Infra-Red Stealth at High Temperature Up to 1300 °C

Mengya Zhu[1, 2, #], Guangyong Li[2, #], Wenbin Gong[3], Lifeng Yan[4], and Xuetong Zhang[2, 5, *]

[1] School of Nano-Tech and Nano-Bionics, University of Science and Technology of China, Hefei 230026, P. R. China

[2] Suzhou Institute of Nano-Tech and Nano-Bionics, Chinese Academy of Sciences, Suzhou 215123, P.R. China

[3] School of Physics and Energy, Xuzhou University of Technology, Xuzhou 221018, P.R. China

[4] School of Chemistry and Materials Science, University of Science and Technology of China, Hefei 230026, P.R. China

[5] Division of Surgery & Interventional Science, University College London, London, NW3 2PF, UK

[#] Mengya Zhu and Guangyong Li contributed equally to this work.

*Corresponding authors. E-mail: xtzhang2013@sinano.ac.cn (Xuetong Zhang), xuetong.zhang@ucl.ac.uk (Xuetong Zhang)

Supplementary Figures

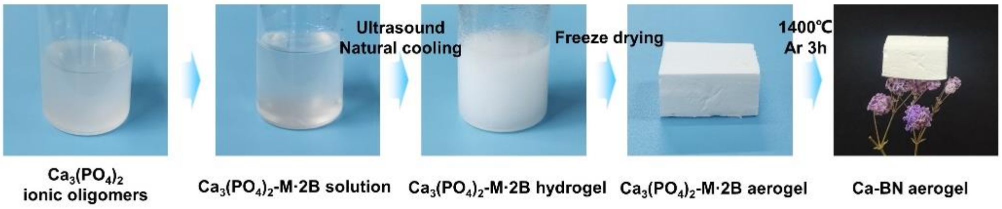

Fig. S1 Photographs of the synthesis of Ca-BN aerogel

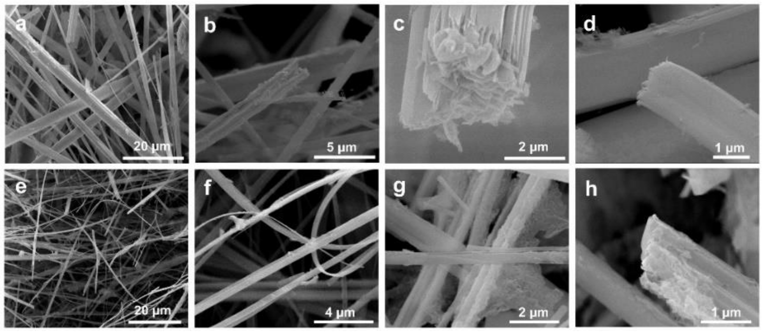

Fig. S2 SEM images of $Ca_3(PO_4)_2$-M·2B aerogel

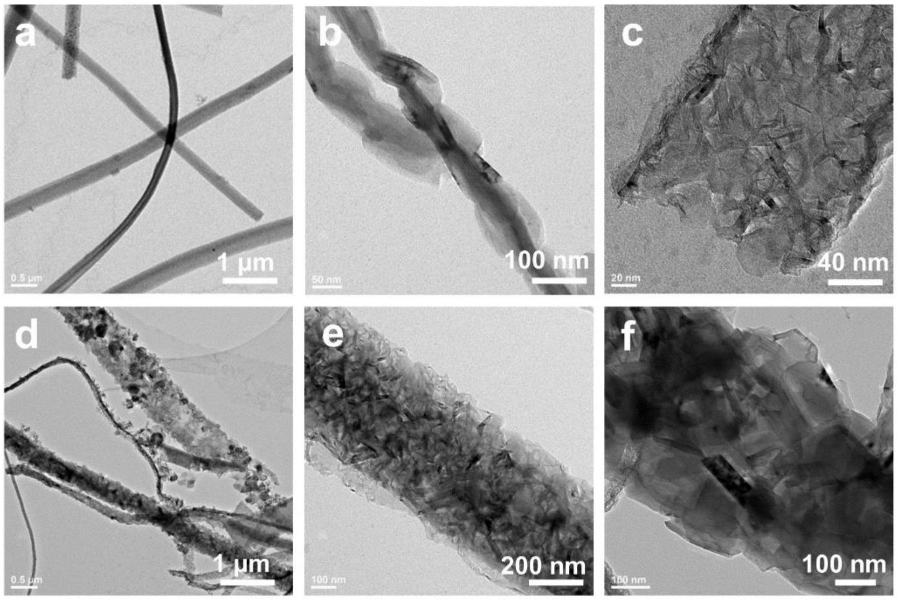

Fig. S3 TEM images of Ca-BN aerogel

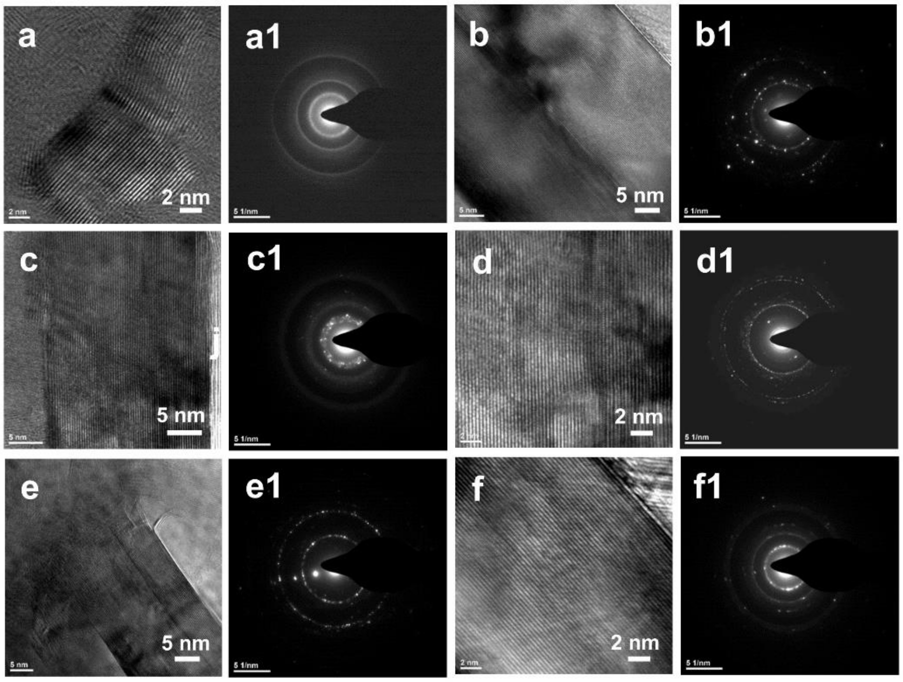

Fig. S4 HRTEM images and the corresponding selected area electron diffraction patterns of the Ca-BN aerogel

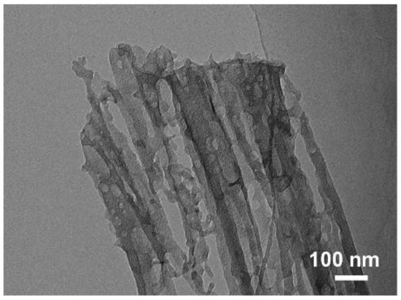

Fig. S5 TEM image of BN aerogel

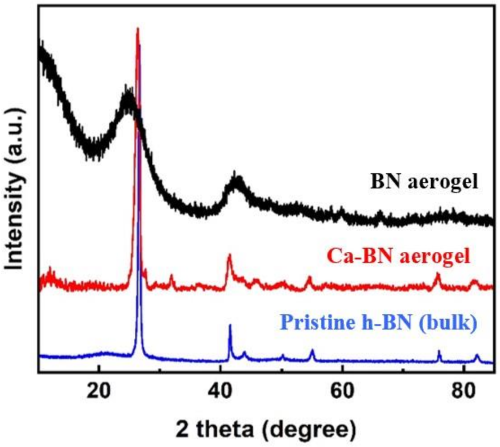

Fig. S6 XRD spectra of Ca-BN aerogel (red), bulk h-BN (blue) and BN aerogel (black). For Ca-BN aerogel, the (002) (peak at 2θ=26.36°) interplanar spacing was about 0.338 nm, which is slightly larger than that of bulk h-BN (0.333 nm, 2θ=26.74°), but is smaller than that of BN aerogel derived from M·2B (0.36 nm, 2θ=24.64°)

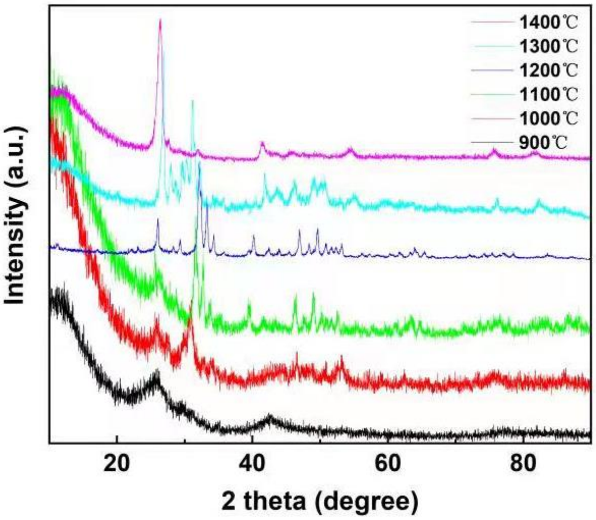

Fig. S7 XRD spectra of Ca-BN aerogels derived at different temperature. During the synthetic process, crystal structure of BN changes from amorphous to crystalline as temperatures increase from 900 °C

to 1400 °C. When synthetic temperature changes from 1000 °C to 1300 °C, other than diffraction peaks of BN, some diffraction peaks of $Ca_3(PO_4)_2$ still were observed in the XRD spectra. When synthetic temperature reaches to 1400 °C, nearly no obvious diffraction peaks of $Ca_3(PO_4)_2$ could be observed, mainly because $Ca_3(PO_4)_2$ occurred carbothermal reduction reaction.

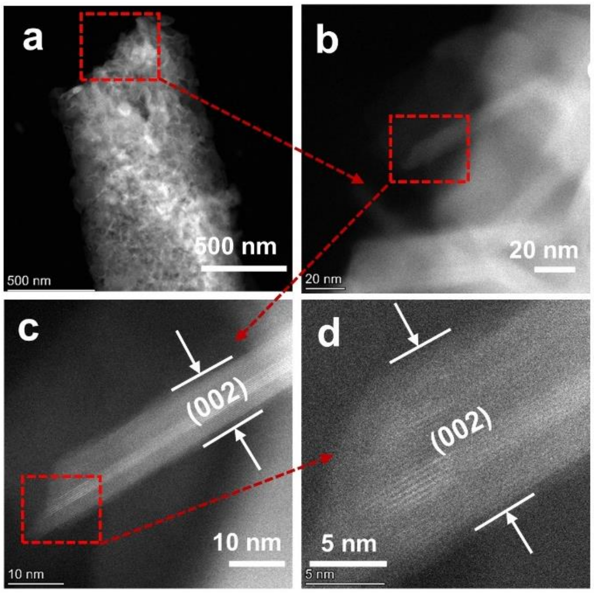

Fig. S8 STEM images of single ribbon of Ca-BN aerogel

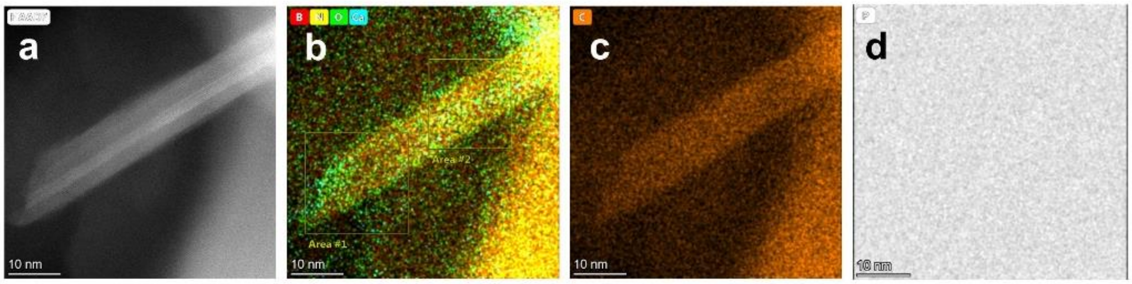

Fig. S9 (**a**) STEM image, (**b**) EDS overlaid mapping of selected elements (B, N, O, Ca), (**c-d**) EDS mapping of C, P of the resulting Ca-BN aerogel in the length of 10-20 nm

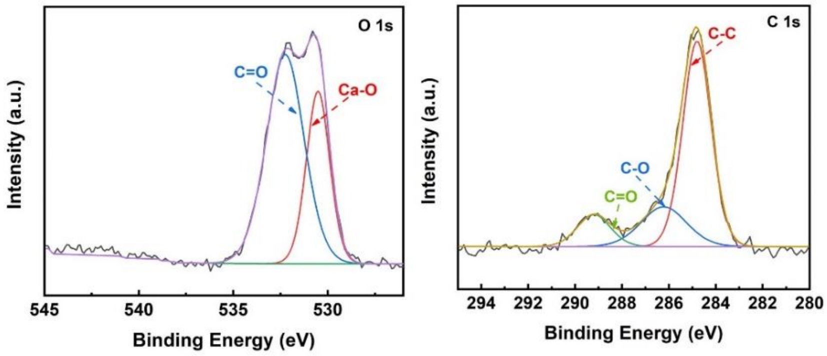

Fig. S10 High-resolution XPS spectra of O 1s and C 1s

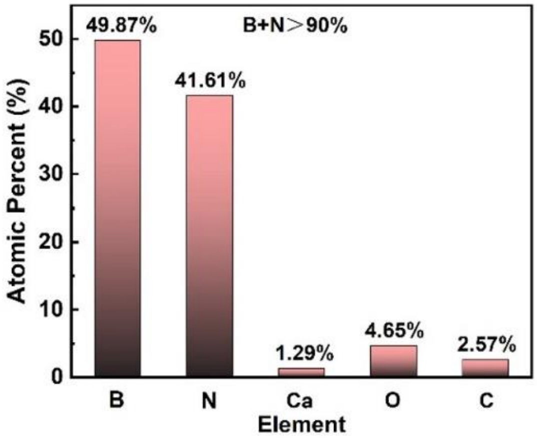

Fig. S11 Atomic ratio of B, N, Ca, O, C from XPS survey. Atomic ratio of B and N is close to 1:1, and total atomic ratio of B and N was about 92.4%, atom ratio of Ca was about 1.29%

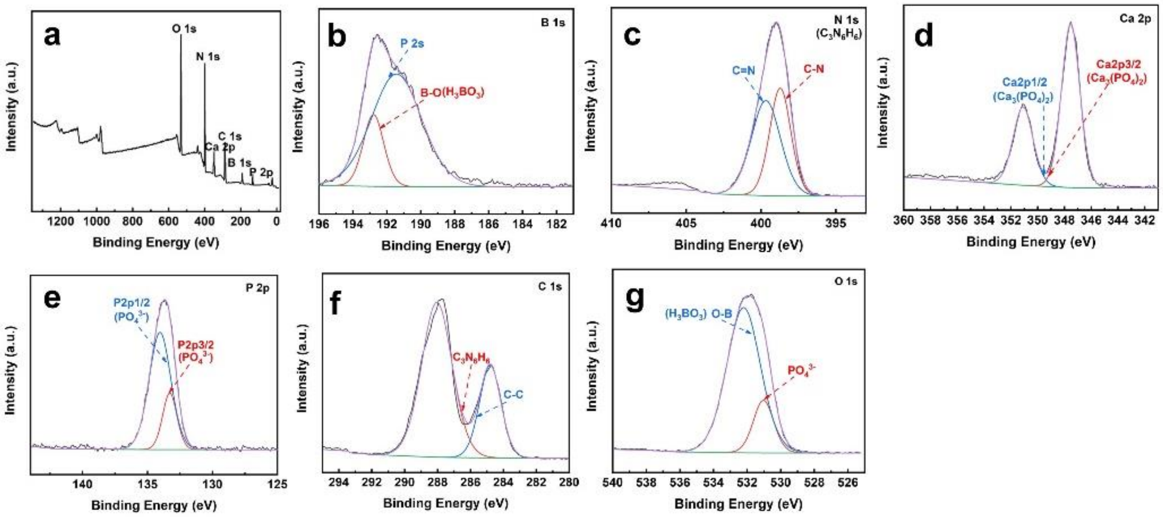

Fig. S12 (**a**) The XPS survey of $Ca_3(PO_4)_2$-M·2B aerogel, (**b-g**) High-resolution XPS spectra of B1s, N1s, Ca 2p, P 2p, C1s, O1s

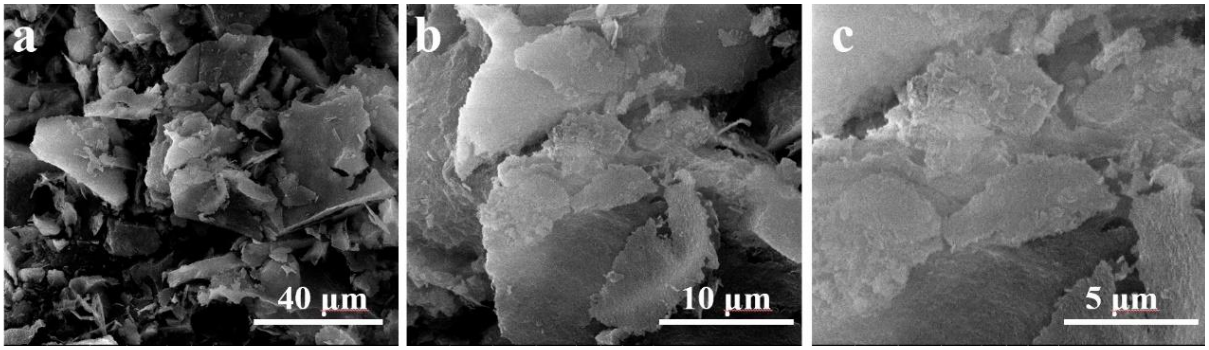

Fig. S13 SEM images of calcium phosphate solid fabricated from calcium phosphate ionic oligomer solution

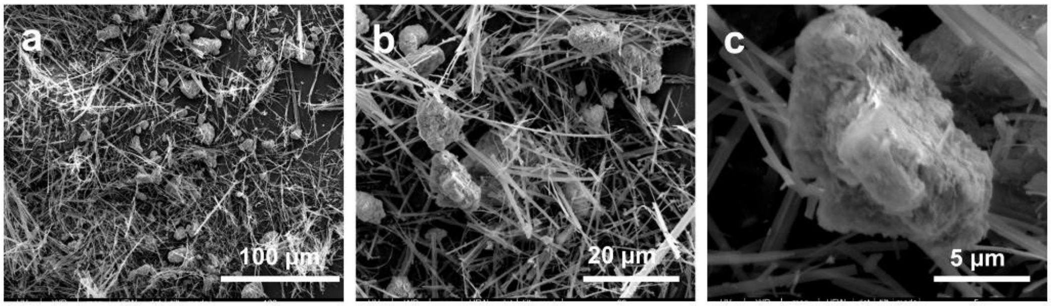

Fig. S14 SEM images of commercial $Ca_3(PO_4)_2$ powder-M·2B aerogel

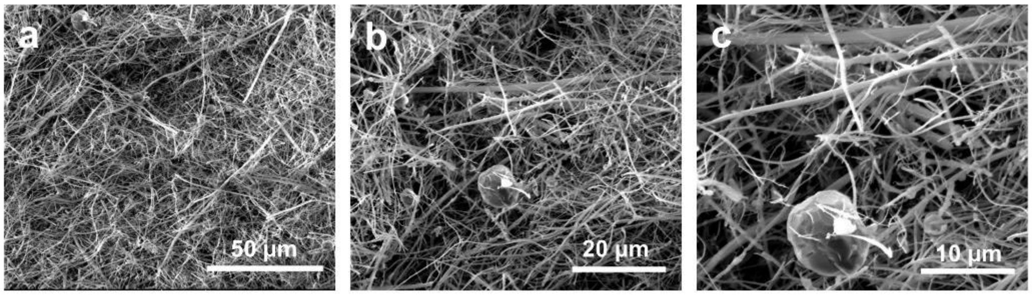

Fig. S15 SEM images of commercial $Ca_3(PO_4)_2$ powder directed Ca-BN aerogel

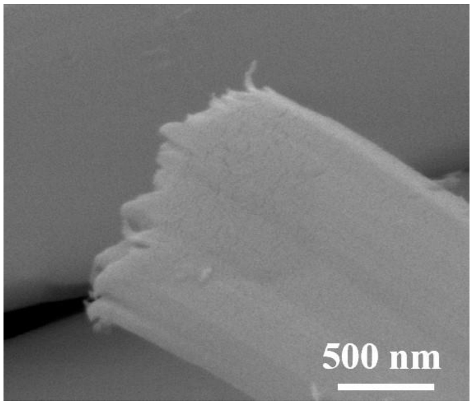

Fig. S16 SEM image of calcium phosphate doped M·2B ribbon

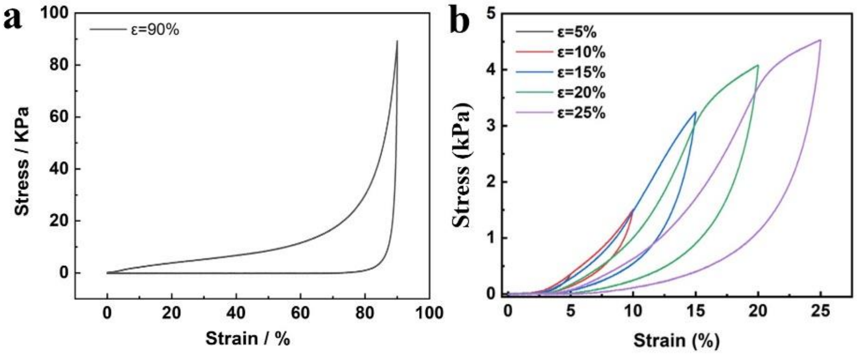

Fig. S17 Strain-stress curves of Ca-BN aerogel

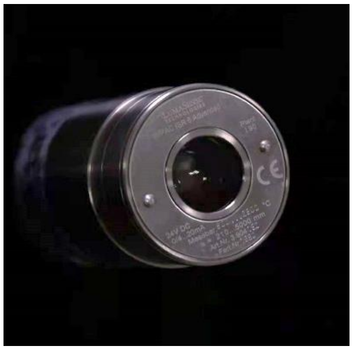

Fig. S18 Photograph of infrared thermometer (ISR 6 Advanced, LumaSense IMPAC)

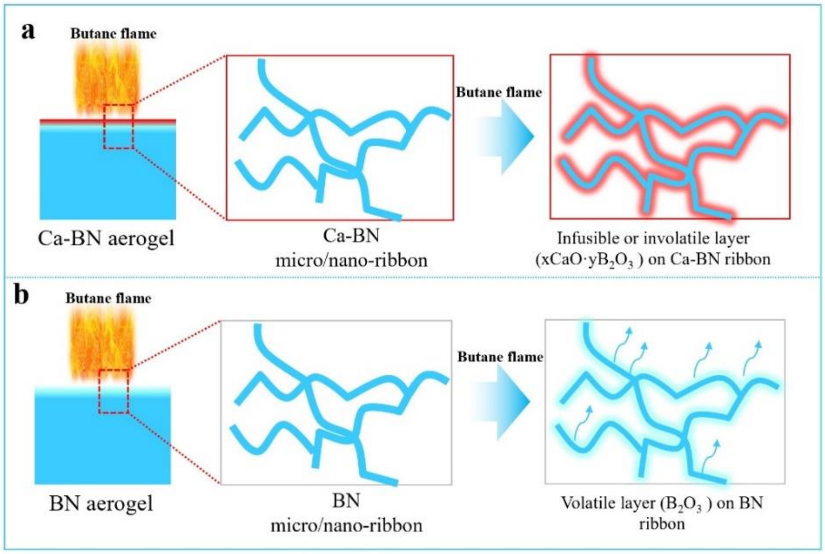

Fig. S19 Illustration of structural evolution of Ca-BN aerogel and BN aerogel when they heated by butane flame. For Ca-BN aerogel, Ca element could react to melted B_2O_3 and then form an involatile melted compound (e.g., $xCaO{\cdot}yB_2O_3$ system), the melted compound covered on the skeleton surface of the aerogel and prevented the further oxidization, pure BN aerogel was oxidized into B_2O_3 and then evaporated rapidly.

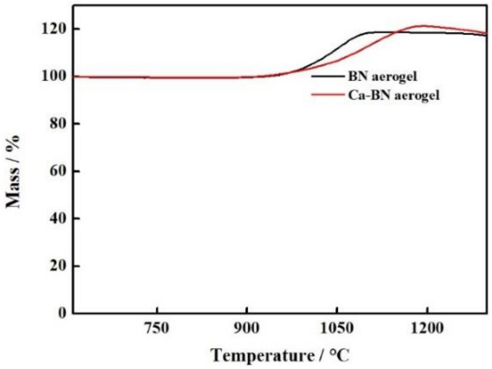

Fig. S20 TG curves of Ca-BN aerogel and BN aerogel in N_2/O_2 atmosphere. Compred to BN aerogel, Ca-BN aerogel exhibited a higher oxidizing temperature.

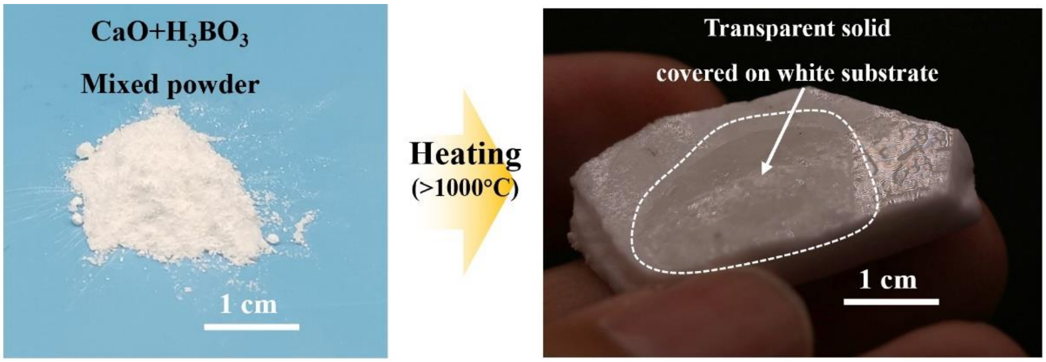

Fig. S21 Photograph of CaO and boric acid and their relevant product after high-temperature treatment

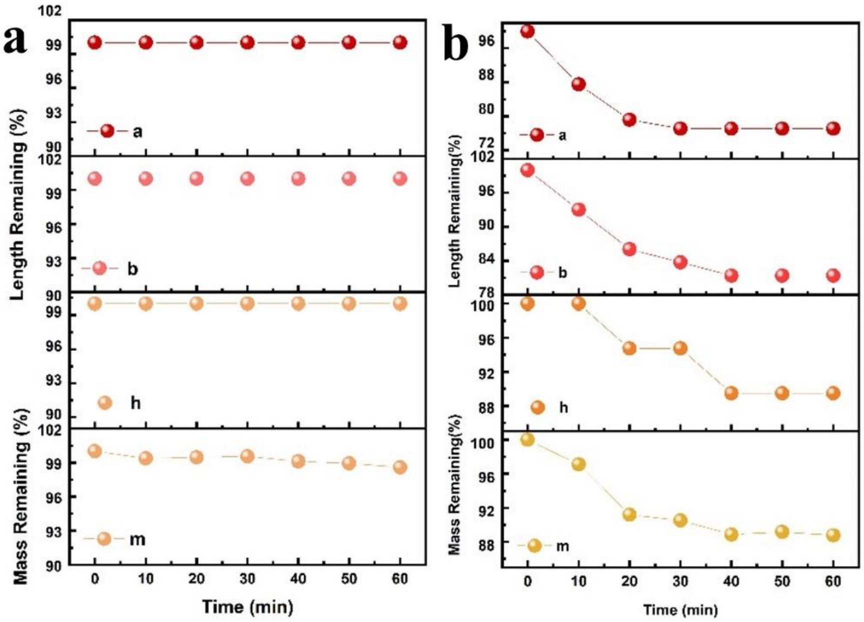

Fig. S22 Statistic diagram of the change rate of three side lengths and mass of Ca-BN (**a**) and BN aerogels (**b**)

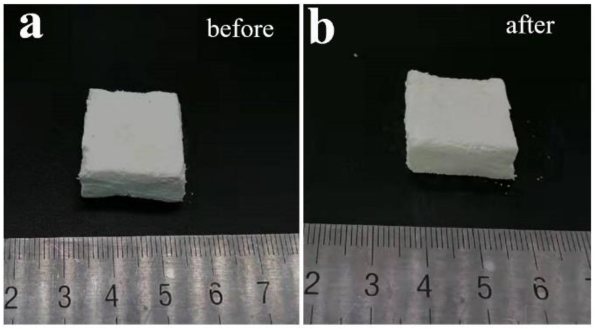

Fig. S23 Surface images of Ca-BN aerogels before (**a**) and after (**b**) being heated by butane flame

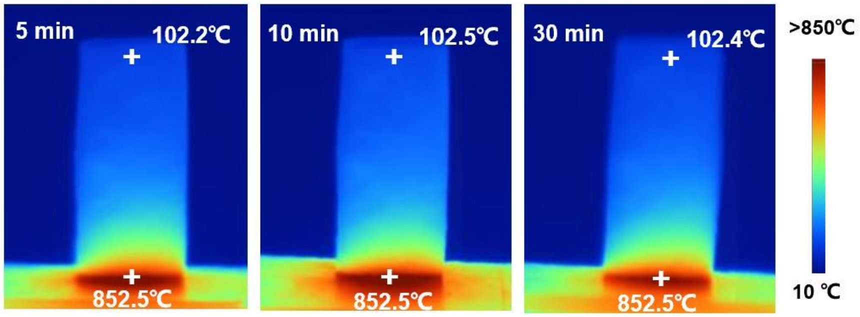

Fig. S24 Infrared images of Ca-BN aerogel on a hot Al_2O_3 plate for 30 min

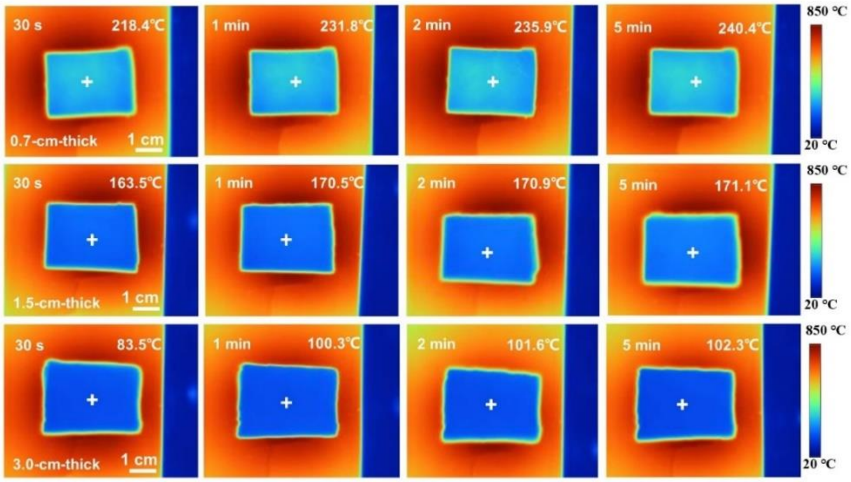

Fig. S25 Infrared images of hot target covered by Ca-BN aerogels with different thickness

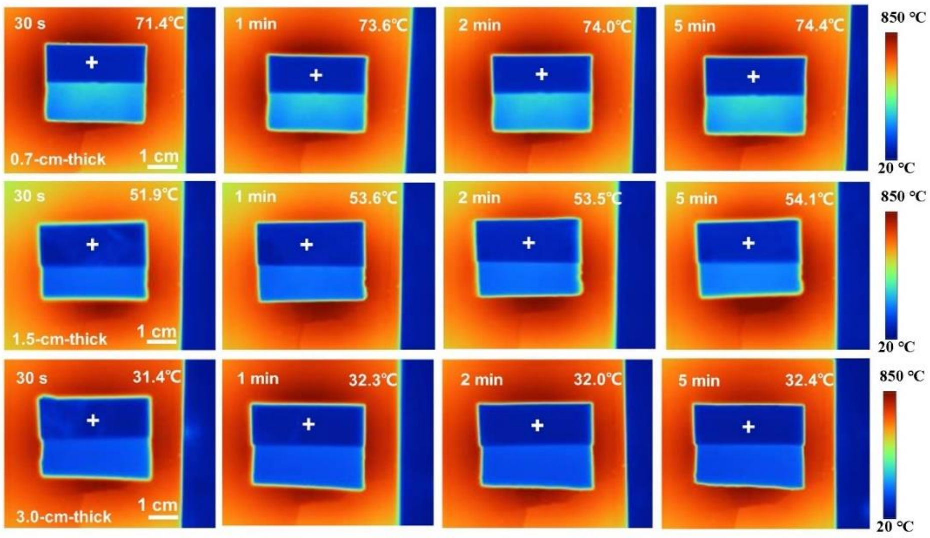

Fig. S26 Infrared images of hot target covered by Ca-BN aerogels (different thickness) together with Al foil

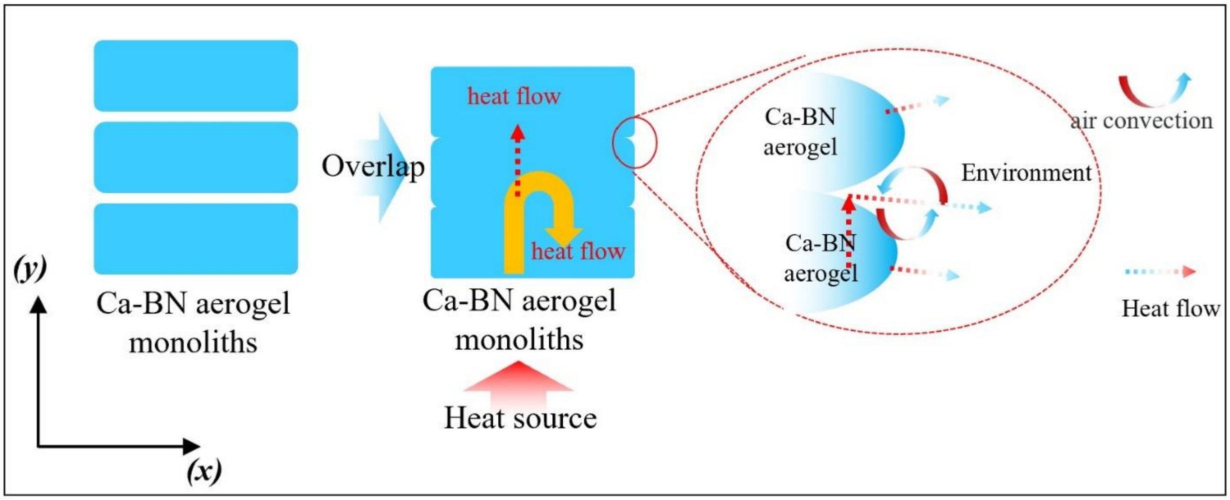

Fig. S27 Illustration of three layers of Ca-BN aerogels. Ca-BN aerogel monoliths were physically stacked together to restrain heat transport in vertical direction (*oy*), aerogel surfaces could touch with each other tightly excepted to their edge positions. At their edge positions, overlapping may bring small gaps because the surface of Ca-BN aerogels cannot be completely flat. Such gaps connected to surrounding environment, which could provide more locations for heat loss from Ca-BN aerogel to environment via air convection in horizontal direction (*ox*). In other words, heat transport along vertical direction (*oy*) was restrained.

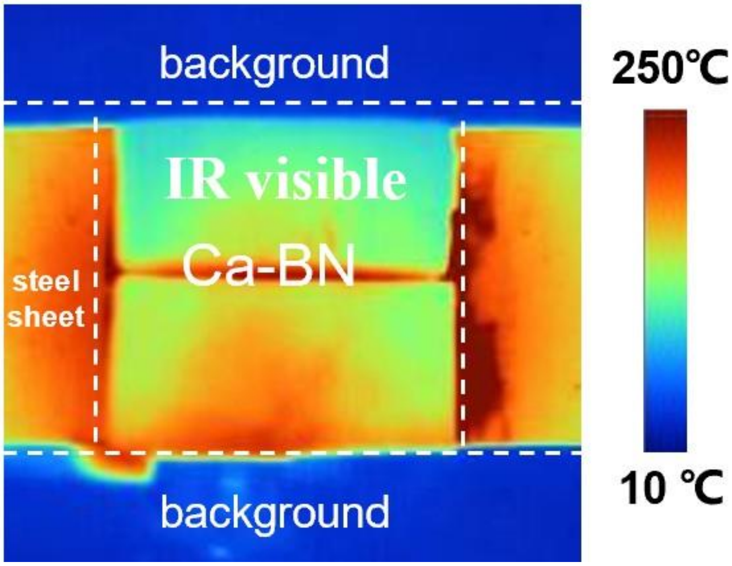

Fig. S28 Infrared image of hot target covered by Ca-BN aerogel
